# Presynaptic antiseizure medications - basic mechanisms and clues for their rational combinations

**DOI:** 10.1007/s43440-024-00603-7

**Published:** 2024-05-22

**Authors:** Ewa K. Czapińska-Ciepiela, Jarogniew Łuszczki, Piotr Czapiński, Stanisław J. Czuczwar, Władysław Lasoń

**Affiliations:** 1 Epilepsy and Migraine Treatment Center, 31-209 Kraków, Poland; 2 Department of Occupational Medicine, Medical University of Lublin, 20-090 Lublin, Poland; 3 Department of Pathophysiology, Medical University of Lublin, 20-090 Lublin, Poland; 4 Maj Institute of Pharmacology, Department of Experimental Neuroendocrinology, Polish Academy of Sciences, 31-343 Kraków, Poland

**Keywords:** Epilepsy, Antiseizure medications, Neurochemical mechanisms, Presynaptic machinery, Clinical efficacy, Rational polytherapy

## Abstract

Among clinically highly efficient antiseizure medications (ASMs) there are modifiers of the presynaptic release machinery. Of them, levetiracetam and brivaracetam show a high affinity to the synaptic vesicle protein type 2 A (SV2A), whereas pregabalin and gabapentin are selective ligands for the α2δ1 subunits of the voltage-gated calcium channels. In this paper, we present recent progress in understanding the significance of presynaptic release machinery in the neurochemical mechanisms of epilepsy and ASMs. Furthermore, we discuss whether the knowledge of the basic mechanisms of the presynaptically acting ASMs might help establish a rational polytherapy for drug-resistant epilepsy.

## Introduction

Epilepsy is a common and multifactorial neurological disorder characterized by a recurrent occurrence of unprovoked convulsive or non-convulsive seizures, which are clinical manifestations of abnormal, transient, and synchronous hyperactivity of specific neuronal circuits in the brain. Depending on the brain region involved in the generation and spreading of seizures, distinct autonomic, motor, and sensory symptoms can be observed [1, 2]. Despite the introduction of new antiseizure medications (ASMs; formerly referred to as antiepileptic drugs), nearly 30% of patients with epilepsy are still resistant to pharmacological treatment [3]. Therefore, both designing new antiepileptic compounds, as well as developing a rational polytherapy using the already marketed ASMs for treating drug-resistant forms of epilepsy seem entirely justified. The maximal electroshock (MES) test, the subcutaneous pentylenetetrazol (scPTZ) seizure test, and the kindling model are animal models routinely used for screening potential ASMs. However, a predictive value of MES and scPTZ is limited to generalized tonic-clonic seizures, while kindling is predictive of human focal epilepsy. Furthermore, the genetic absence epileptic rat of Strasbourg and the lethargic (lh/lh) mouse are predictive of human generalized spike-wave seizures [4]. It is emphasized that no single model has a predictive value for drug-resistant seizures, but rather a battery of such models should be used [5]. Recently, more etiologically relevant models in the preclinical evaluation of new investigational drugs for the treatment of drug-resistant epilepsy have been comprehensively reviewed [6].

Neurochemical and electrophysiological studies show that seizures result from brain region-specific excessive excitatory processes or insufficiency of inhibitory neurotransmission. Thus, the hyperactivity of the glutamatergic neurons leads to excessive activation of the ionic excitatory amino acid receptors, e.g. the N-methyl-D-aspartate (NMDA), α-amino-3-hydroxy-5-methyl-4-isoxazolepropionic acid (AMPA), and kainate receptors, resulting in depolarization of neurons and facilitation of epileptic phenomena [7, 8]. Furthermore, alterations in expression, loss- or gain-of-function mutations of protein subunits, polymorphisms, and cellular energetic deficits can all contribute to dysfunction of the voltage- and ligand-dependent sodium, calcium, potassium, and chloride channels, eventually promoting seizure discharges [9]. The γ-aminobutyric acid (GABA) is regarded as the principal inhibitory neurotransmitter in the mammalian brain. It has been well evidenced that even a moderate inhibition of the chloride ion-gated GABA_A_ receptors can provoke seizures. The deficit in the inhibitory processes is predominantly linked with insufficiency in GABA_A_ receptor-mediated neurotransmission [10].

Accordingly, pharmacological strategies in the treatment of epilepsy include stabilization of neuronal membranes and preventing depolarization by acting on ion channels, increasing and decreasing the GABAergic and excitatory amino acid (EAA) transmission, respectively. In this paper, we review the recent progress in understanding the neurochemical and molecular mechanism of presynaptic release machinery as targets for ASMs. Furthermore, we discuss whether the knowledge of the neurochemical mechanisms of the ASMs - with a special emphasis on racetams and gabapentinoids - might help establish a rational polytherapy for drug-resistant epilepsy.

### Mechanisms of action of current ASMs

**Fig. 1:**
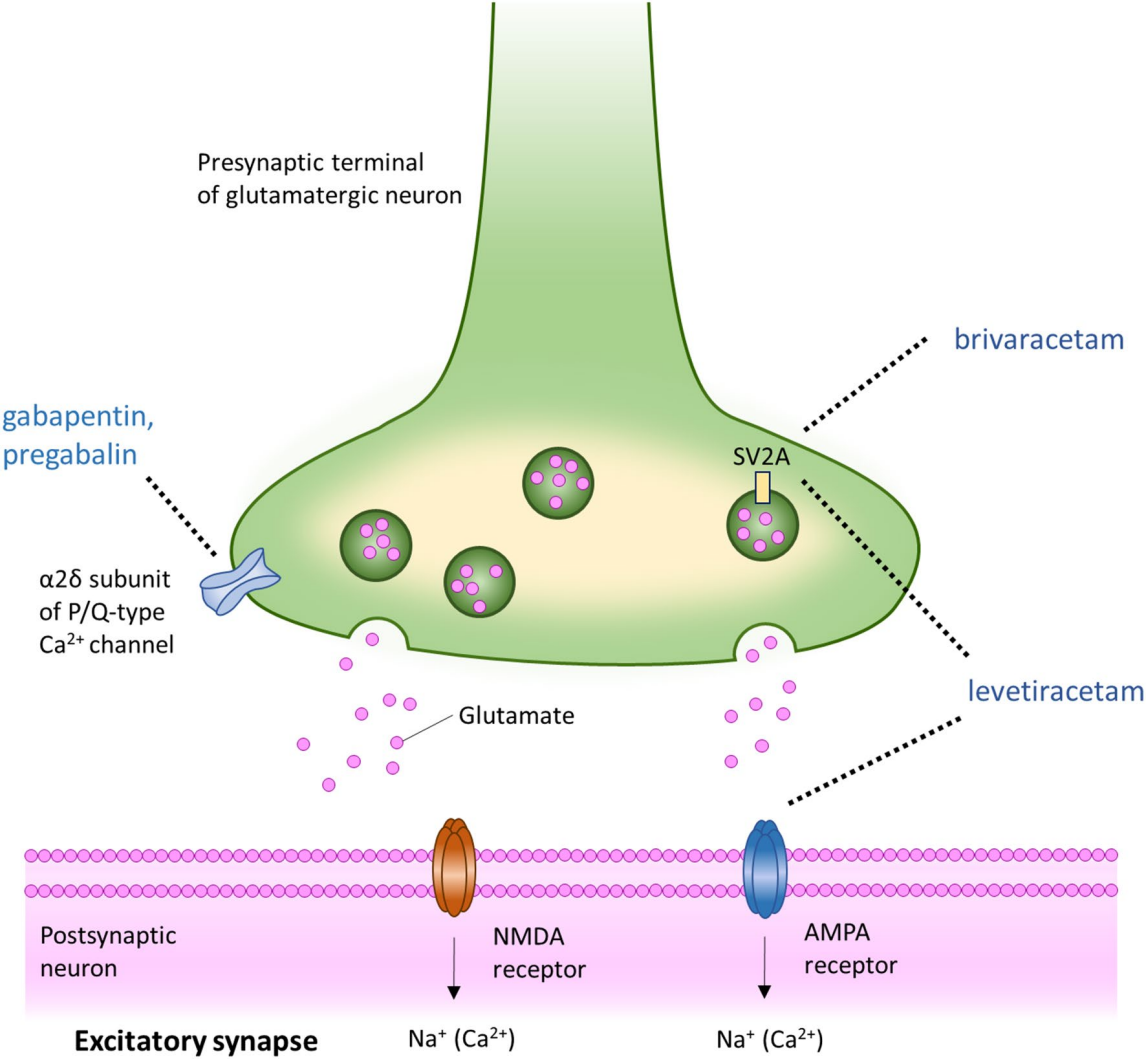
The main neuronal targets of racetams and gabapentinoids (inspired by the figure in the paper by Bialer and White [166])

Current ASMs have diverse mechanisms of action. Some of them block voltage-gated Na^+^ channels (VGSCs) or voltage-gated Ca^2+^ channels (VGCCs), increase GABA concentrations, block glutamate receptors, inhibit carbonic anhydrase, activate GABA_A_ receptors (GABA_A_R) r or modulate synaptic vesicles [11]. Most of the ASMs are multitargeted drugs, although in each drug one of the neurochemical mechanisms appears to predominate. Of the marketed ASMs, phenytoin, primidone, carbamazepine, oxcarbazepine, eslicarbazepine, lamotrigine, rufinamide, and zonisamide bind to VGSCs in their state of fast inactivation (within milliseconds) and induce transient, voltage- and frequency-dependent reduction in the channel conductance [12]. Lacosamide was reported to block slow inactivation (within seconds and beyond) of VGSCs through binding to sodium channel in its state of slow inactivation and inducing its long-lasting, voltage- and frequency-dependent reduction in the channel conductance. Alternatively, its mode of action can be linked to its slow dissociation from the target molecule [13]. Reduction of low-threshold calcium current in thalamic neurons by ethosuximide or sodium valproate prevents synchronized depolarization, particularly in thalamic-cortical circuits [14]. Among ASMs that recover the balance between the excitatory glutamatergic and inhibitory GABAergic transmission on the receptor levels are phenobarbital (a positive allosteric modulator of the GABA_A_ receptor and an antagonist of the glutamatergic AMPA receptors), felbamate (an antagonist of the NMDA receptors and an allosteric positive modulator of the GABA_A_R), perampanel (a selective non-competitive antagonist of the glutamatergic AMPA receptors) and clobazam (a positive allosteric modulator of the GABA_A_R. Cenobamate is a positive allosteric modulator of the high-affinity GABA_A_R, and it blocks persistent rather than transient sodium currents. ASMs that enhance the synaptic GABA level, include vigabatrin and tiagabine. Vigabatrin irreversibly inhibits GABA-aminotransferase (transaminase) - the enzyme responsible for the metabolic degradation of GABA - leading to a global increase in brain GABA concentration. Tiagabine inhibits the re-uptake of GABA from the synaptic cleft into nerve terminals and glial cells by selective action on the GAT-1 transporter, resulting in a transient increase in synaptic GABA levels. ASMs with complex and only partially recognized mechanisms of action comprise valproate (a voltage-gated sodium channel blocker, an inhibitor of the GABA degradative enzymes and an inhibitor of GABA re-uptake), topiramate (a modulator of the voltage-dependent sodium channels, an enhancer of GABA inhibition, an inhibitor of excitatory neurotransmission, an inhibitor of carbonic anhydrase and possibly a modulator of the voltage- and receptor-gated calcium ion channels) and stiripentol (a positive allosteric modulator of the GABA_A_R which also interferes with GABA reuptake and metabolism) [15, 16]. Carbonic anhydrase inhibitors, such as acetazolamide, topiramate, and zonisamide, restore the equilibrium among CO_2_, H^+^, and HCO_3_^−^_,_ reduce the NMDA-mediated excitation, potentiate the GABA_A_R- mediated inhibition, and affect the activity of ligand-gated Ca^2+^ channels and voltage-gated Ca^2+^ channels (VGCC) [17, 18]. There are also several ASMs with predominant mechanisms of action that are not related to modulation of the voltage- and ligand-gated sodium and calcium channels or to enhancement of the GABA brain concentration, such as everolimus (an inhibitor of the mTORC1), fenfluramine (a serotonergic 5-HT2 receptor agonist and a σ1 receptor antagonist) and cannabidiol. Cannabidiol has a complex and partly unknown mechanism of antiseizure effects. It probably reduces neuronal hyper-excitability through modulation of intracellular calcium via G protein-coupled receptor 55 (GPR55), transient receptor potential vanilloid 1 (TRPV-1) channels, and upregulation of the peroxisome proliferator-activated receptor gamma (PPAR-γ) [19, 20]. ASMs inhibit or prevent seizures, but they neither cure epilepsy nor prevent epileptogenesis. Since epilepsy and epileptogenesis seem to be connected with the pathological transformation of neuronal circuits [21], drugs that interfere with molecular mechanisms of synaptic plasticity merit special attention [22]. Two classes of ASMs with unique mechanisms of action are racetams (levetiracetam, brivaracetam) and gabapentinoids (gabapentin, pregabalin), which modulate the neurotransmitter release via binding to glycoprotein 2 A (SV2A) in synaptic secretory vesicles and α2δ subunits of presynaptic voltage-gated calcium channels, respectively [15]. Figure 1.

### Presynaptic release machinery as targets for ASMs

Since an imbalance in excitatory versus inhibitory neurotransmission is thought to be the key player in the pathophysiology of seizures, it implies that the presynaptic release machinery of these neurons could serve as an important target for ASMs and possibly for designing epilepsy-modifying drugs. The molecular presynaptic events include the neurotransmitter cycle (biosynthesis, storage, reuptake, and degradation of neurotransmitters) and the synaptic vesicle cycle (targeting synaptic vesicles (SVs) to the nerve terminal and their docking, fusion, endocytosis, and recycling) [23]. Each of these processes in the overlapping presynaptic cycles may affect the mode and the amount of neurotransmitter released. Both GABA and glutamate accumulate in SVs, which undergo exocytosis within the active zone of the synapse. High rates of exocytosis depend on the efficient packaging of neurotransmitter into SV and the fast recycling of SV from the presynaptic plasma membrane or via an endosomal intermediate. The SV recycling comprises sorting of its proteins from proteins of the plasma membrane, clathrin-regulated endocytosis, and docking of SV at the plasma membrane. Activation of presynaptically localized voltage-gated calcium channels leads to a local elevation of Ca^2+^ in the nerve terminal which regulates vesicle fusion [24]. Several proteins that are constituents of the SV cycle have been identified and their role partially unraveled [25]. Among SV proteins involved in targeting and docking are synapsins which link SVs to the actin cytoskeleton, piccolo and bassoon engaged in active zone assembly, and the Sect. 6/8 complex which defines the site of the active zone. Several synaptic proteins such as synaptobrevin, vesicle-associated SNARE (v-SNARE), and two target or plasma membrane SNAREs (t-SNAREs), synaptosomal associated protein 25 (SNAP25) and syntaxin form a stable complex that is probably engaged in both SV docking and fusion [26]. Proteins that are involved in modulation and Ca^2+^ dependence of the synaptic vesicle cycle are represented by C1 and C2 domain protein (Munc13) required for neurotransmitter release, N, and P/Q-type Ca^2+^ channels, Ca^2+^ sensor (synaptotagmin) C2 domain SV protein; and SV2 transporter which inhibits SV fusion. The synaptic vesicle glycoprotein 2 A is selectively expressed in the vesicular membranes of neuronal terminals and - via the modulation of the calcium sensor - synaptotagmin-1 activity - it regulates the Ca^2+−^dependent synaptic exocytosis. It may be engaged in the stabilization of vesicular loading of neurotransmitters, vesicular transporting, vesicular proteins anchoring, and vesicle trafficking [27, 28]. To better understand the role of SV2A in neurotransmitter release, Bradberry and Chapman (2022) used a new chemogenetic approach for all-optical monitoring of excitation-secretion coupling in knockout (KO) phenotype in cultured hippocampal neurons. This method allowed for the detection of the presynaptic Ca^2+^ influx and glutamate release at the same axonal boutons. The authors showed that a loss of SV2A decreased glutamate release without reducing the Ca^2+^ influx at the hippocampal nerve terminals. These data indicate that SV2A, which is trafficked to synaptic vesicles, supports vesicle fusion by increasing the efficiency of the Ca^2+−^regulated membrane fusion machinery [29]. Accumulating data indicate that malfunctioning of the presynaptic release machinery plays an important role in the pathomechanisms of seizures [30]. Hence, disturbances of mechanisms regulating the physiological synergy among presynaptic and postsynaptic components due to mutations of genes encoding some synaptic proteins lead to developmental and epileptic encephalopathies, known as “synaptopathies” [31]. Of them, mutation of the CPLX1 gene encoding the complexin 1 which interacts with the SNARE complex, the STXBP1 gene which encodes the crucial for synaptic exocytosis Syntaxin1a binding protein, and the PRRT2 gene which encodes the presynaptic proline-rich transmembrane protein 2 (Prrt2) are associated with epileptic symptoms [31]. As far as the preclinical research is concerned, convincing evidence for the role of the presynaptic neurotransmitter release machinery in seizures comes from studying Tetanus neurotoxin. Thus, an intracortical injection of Tetanus neurotoxin which cleaves the synaptic protein VAMP/synaptobrevin induces pharmacoresistant and refractory focal cortical hyperexcitability and electrographic seizures in rodents [32, 33]. Furthermore, Vannini et al. (2020) showed in the mouse model of neocortical epilepsy induced by Tetanus neurotoxin an early-onset lengthening of active zones at inhibitory synapses and a delayed spatial reorganization of recycled vesicles at excitatory synapses [34]. Regarding other presynaptically localized proteins, the role of dynamin in clathrin-mediated endocytosis and synaptic vesicle recycling in epilepsy has been postulated [35]. Dynamin 1 (DNM1) is a guanosine triphosphatase engaged in clathrin-mediated endocytosis, and de novo mutations in synaptic transmission genes including DNM1 were reported to be a causative factor in epileptic encephalopathies [36]. Other investigators found that the dynamin 1 expression pattern was altered in the pilocarpine rat model of epilepsy and patients with temporal lobe epilepsy. Moreover, in the above study, inhibition of dynamin diminished seizures [37]. More recently, a new class of GTP-competitive dynamin inhibitors which inhibit dynamin I GTPase and clathrin-mediated endocytosis showed a significant anti-seizure activity in a 6 Hz mouse psychomotor seizure test [38]. The role of Syntaxin 7 (STX7) - a member of the SNARE superfamily involved in membrane fusion - was investigated in the models of kainate-induced seizures and PTZ-induced kindling. In both models of epilepsy, the STX7 expression was decreased in the brain tissue. It was also observed that overexpression of STX7 alleviated seizures, while the downregulation of this protein evoked opposite effects [39]. The above-cited reports suggest that some proteins involved in presynaptic release machinery, such as dynamin and STX7, may be considered targets for novel ASMs, but future studies should show which of these proteins may be druggable. Unsurprisingly, more extensive studies were devoted to the engagement of SV2A in the pathomechanism of seizures, since this protein is an established target for the already marketed ASMs. It has been shown that the dysfunction of SV2A which regulates the action potential-dependent neurotransmitter release may be involved in epileptogenesis since SV2A expression in the brain was elevated specifically in the dentate hilus in PTZ kindling and down-regulated in the anterior temporal neocortex in patients with intractable temporal lobe epilepsy and focal cortical dysplasia [40]. On the other hand, the physiological level of SV2A prevents seizures, because the homozygous sv2a knockout leads to a lethal seizure phenotype in mice, probably resulting from an imbalance between the inhibitory and excitatory neurotransmission [41]. This hypothesis has been supported by electrophysiological studies which demonstrated that sv2a deletion reduces the action of the potential-dependent release of GABA in the CA3 region of the hippocampus [41], while the cultured hippocampal neurons from sv2a/2b double knockouts display a sustained increase in the excitatory calcium-dependent synaptic neurotransmission [42]. Also, another body of evidence points to a possible role of SV2A in epileptogenesis. To this end, the microarray study on ipsilateral hippocampal CA3 and entorhinal cortex showed that sv2a was transiently downregulated in the entorhinal cortex during pilocarpine-induced epileptogenesis in rats [43]. Furthermore, van Vliet et al. (2009) conducted a comparative study on the expression of SV2A protein in resected temporal lobe specimens from patients with refractory epilepsy and in the hippocampi of rats subjected to experimental epileptogenesis by tetanic stimulation of the angular bundle. They found that the SV2A immunoreactivity was decreased in all specimens from patients with confirmed hippocampal sclerosis, whereas the SV2A protein expression in the hippocampus of rats in the chronic epileptic phase was also reduced, but only in those animals with a progressive form of epilepsy [44]. The authors suggested that a decreased expression of SV2A did not cause immediate seizures but could contribute to a state of heightened epileptogenicity. This assumption has been supported by the study performed by Kaminski et al. (2009), who showed that SV2A (+/−) heterozygous mice had a high sensitivity to seizures evoked by kainate, pilocarpine, PTZ, and 6-Hz electrical stimulation, but no such sensitivity was seen in the model of MES. The aforementioned investigators also observed that SV2A (+/−) heterozygous mice showed accelerated epileptogenesis in the amygdala and corneal kindling models, suggesting that even a partial SV2A insufficiency may accelerate epileptogenesis and increase the vulnerability to seizures [45]. Interestingly, studies using animals with the Sv2a missense mutation revealed that the dysfunction of SV2A preferentially disrupts the action potential-induced GABA, but not glutamate, released in the hippocampus and amygdala and facilitates kindling, which points to the important role of SV2A in GABA neurotransmission [40]. Loss of SV2 affects the calcium-stimulated exocytosis and lack of SV2 proteins, presynaptic calcium accumulation during consecutive action potentials produces abnormal enhancement in neurotransmitter release that destabilizes the synaptic circuits and evokes seizures [42]. As pointed out by Ciruelas et al. (2019), since SV2A is the primary SV2 paralog in the majority of GABAergic neurons, reductions in its expression could potentially contribute to the etiology of epilepsy and that precise control of SV2A expression is needed to prevent aberrant excitability [46]. The intriguing hypothesis addressing a possible relationship between a lowered SV2A expression and the progressive nature of the resultant epileptic state has been questioned and needs further evidence [47].

Kaminski et al. (2008) demonstrated that there is a strong correlation between the SV2A binding affinity of structurally diverse ligands and their anticonvulsant potency in vivo [48]. This hypothesis might contradict the fact that the absence of SV2A in mice led to an epileptic phenotype [41], but the elevated expression of SV2 was also reported to result in a neurotransmission phenotype that resembled that seen in SV2 KO neurons [49] suggesting that either too little or too much SV2A activity may promote neurological abnormality [27]. Furthermore, although SV2A is localized at both inhibitory and excitatory synapses, and the expression of SV2A is ubiquitous, stronger associations between SV2A and GABAergic rather than glutamatergic synapses were observed in some brain structures [50]. SV2A regulates the size of the readily releasable pool of vesicles in the majority of GABAergic neurons, and it has been suggested that reductions in its expression could potentially contribute to the etiology of epilepsy [46]. It has been postulated that reduced expression of SV2A in the hippocampus does not cause immediate seizures but it could contribute to the increased epileptogenicity [44]. On the other hand, pilocarpine-induced seizures– a well-validated model of temporal lobe epilepsy– lead to overexpression of SV2A, which can be normalized by brivaracetam. Bivaracetam treatment ameliorated the over-expression of SV2A in the hippocampus and rescued the synaptic dysfunction in epileptic rats [51]. This drug has been found to modulate short-term synaptic potentiation and abnormal low-frequency brain activities during the interictal phase of epileptic seizures by slowing down the mobilization of synaptic vesicles [52]. Overall, it seems safe to assume that racetams „normalize” SV2A functions which is in line with the view that both SV2A overexpression and SV2A deficits seem to have negative effects on neurotransmission and excitability [53].

### Neurochemical mechanisms of levetiracetam and brivaracetam

The presynaptic inhibitory effect of levetiracetam on the activity-dependent glutamate and GABA release from rat brain slices, the said effect occurring in a use-dependent manner, was reported [54]. Lynch et al. (2004) first provided several lines of evidence that the synaptic vesicle protein SV2A was the binding site for levetiracetam ((S)-alpha-ethyl-2-oxo-pyrrolidine acetamide). They demonstrated that the levetiracetam binding site was enriched in synaptic vesicles, established its apparent molecular weight, and found that synaptic vesicles from homozygous SV2A knockout mice did not bind labeled levetiracetam. They also observed that levetiracetam bound to SV2A, but not to SV2B and SV2C isoforms expressed in the fibroblasts. Moreover, a strong correlation was found between the affinities of the above compound for SV2A and its antiseizure efficacy in the audiogenic animal model of epilepsy [55]. The anticonvulsant efficacy of levetiracetam was also reduced in heterozygous SV2A+/- mice (the expression of the SV2A protein was decreased by 50%), which lends further support to the notion that SV2A is the primary target for seizure protection [45]. Brivaracetam was identified as a result of screening of 12,000 compounds for binding affinity to the synaptic vesicle protein 2 A (SV2A). Brivaracetam binds selectively in a reversible, saturable, and stereospecific fashion - and with a 20-fold higher affinity than levetiracetam - to SV2A in rat and human brains, and no specific binding was detected in the brain of SV2A(-/-) knock-out mice. The effects of brivaracetam on the synaptic plasticity in experimental models of epilepsy have been demonstrated. Thus, chronic administration of brivaracetam ameliorated the over-expression of SV2A and the synaptic dysfunction in the pilocarpine model of temporal lobe epilepsy in rats. These effects were accompanied by normalization of fast phosphorylation of the synaptosomal-associated protein 25 (SNAP-25) during long-term potentiation in the epileptic rats, which may be relevant to the modulation of SV exocytosis and activity of voltage-gated calcium channels [51]. Based on its affinity and pharmacokinetic parameters, it is predicted that at therapeutic concentrations, brivaracetam should occupy over 80% of SV2A in the human brain [56]. Brivaracetam decreased the short-term synaptic potentiation and abnormal low-frequency brain activities during the interictal phase of epileptic seizures by slowing down the mobilization of synaptic vesicles in two rodent models of epilepsy evoked by pilocarpine or high potassium concentrations [52]. Repeated administration of brivaracetam reduced the over-expression of SV2A. It alleviated the abnormal SNAP-25 phosphorylation at Ser187 during long-term potentiation (LTP) induction in epileptic rats, which is relevant to the modulation of synaptic vesicle exocytosis and voltage-gated calcium channels. The exact mechanism of anti-seizure effects of SV2A ligands has not been elucidated [15]. It has been presumed that levetiracetam reduces the excitatory neurotransmitter release during epileptic high-frequency activity by accessing its binding site through vesicular endocytosis into excitatory synaptic terminals. However, as shown by patch-clamp recording, levetiracetam evoked a similar, frequency-dependent effect on both inhibitory postsynaptic currents (IPSCs) and excitatory postsynaptic currents (EPSCs) in the hippocampal neurons; thus, its effects on pathological discharges remain yet to be elucidated [54]. Contreras-García et al. (2022) discussed the main hypothesis referring to the effect of levetiracetam on SV2A. Thus, blocking of SV2A by levetiracetam may prevent the SV2A-induced vesicular priming, decrease the size of the readily releasable pool of neurotransmitter and decrease the synaptic transmission. Alternatively, levetiracetam could stabilize the best functional conformation of SV2A and improve synaptic vesicle exocytosis [57]. Both SV2A overexpression and SV2A deficits exert negative effects on neurotransmission. Treatment with levetiracetam was shown to reestablish normal neurotransmission and restore the normal levels of SV2 and synaptotagmin in the overexpressed SV2A synapses [49]. Of note, levetiracetam is regarded as a multitargeted drug because - apart from interacting with SV2A protein - it triggers several other pre- and postsynaptic effects. At the presynaptic level, it reduces the potassium currents, and calcium transients of ryanodine and inositol 1,4,5-trisphosphate receptor (IP_3_R), modulates the level of glutamic acid decarboxylase, and increases the activity of GABA transaminase (GABA-T). At the post-synaptic level, it modulates the AMPA receptors [57]. A radioligand binding study revealed that levetiracetam and brivaracetam may interact differently with the SV2A protein binding sites or interact with different conformational states of this protein [58]. Furthermore, a recent electrophysiological patch-clamp study showed that brivaracetam dose-dependently inhibited the depolarization-induced M-type K^+^ current (IK(M)), decreased the delayed-rectifier K^+^ current (IK(DR)), decreased the hyperpolarization-activated cation current and had a concentration-dependent inhibitory effect on the voltage-gated Na^+^ current (INa) in the GH3 neurons. It is suggested that brivaracetam may have a multiple ionic mechanism of action in disorders linked to neuronal hyperexcitability [59]. However, in contrast to carbamazepine, brivaracetam did not affect sustained repetitive firing in cultured cortical neurons and in CA1 neurons, which indicates that its effect on the voltage-gated sodium channels does not contribute to its antiepileptic activity [60]. Using the SV2A knockout model in zebrafish, Zhang et al. (2022) observed that homozygous SV2A -/- mutant zebrafish larvae, but not SV2A +/- and SV2A +/+ larvae, showed a spontaneous epileptiform activity without brain malformations. Both valproate and, surprisingly, levetiracetam partially reduced the epileptiform activity, which suggests that besides SV2A, other mechanisms may be involved in the antiepileptic effects of levetiracetam [61]. As far as the tripartite synaptic transmission is concerned, it has been reported that levetiracetam and brivaracetam - through the inhibitory effect on SV2A - suppress the glutamate release from astrocytes during high-frequency oscillation bursts. The authors suggest that the suppression of SV2A function and the subsequent inhibition of turnover prolongation of the activated astroglia hemichannels may contribute to the mechanism of anti-seizure effects of levetiracetam and brivaracetam [62, 63]. No data have been found in PubMed that would indicate that ASMs other than racetams have been tested regarding their affinities to SV2A. However, padsevonil, an antiepileptic drug candidate with a high affinity for SV2A, SV2B, and SV2C isoforms and low-to-moderate affinity for the benzodiazepine binding site on GABA_A_ receptors, has been recently designed [64].

### Pharmacological profiles of levetiracetam and brivaracetam in rodent models of seizures and epilepsy

Levetiracetam shows a high anti-seizure activity in electrically and PTZinduced kindling in mice, as well as in pilocarpine- and kainic acid-induced secondary generalized seizures in mice and rats. In contrast, levetiracetam was ineffective in the acute MES test, in the maximal PTZ seizure test in mice, and in several maximal chemoconvulsive seizure tests (Table 1). Furthermore, levetiracetam displayed a very high safety margin in animal models of seizures [65]. Matagne et al. (2008) conducted a comparative in vitro and in vivo study of the anti-seizure activity of brivaracetam and levetiracetam. They reported that brivaracetam was more potent than levetiracetam in reducing epileptiform responses in rat hippocampal slices. As opposed to levetiracetam, brivaracetam protected mice against MES or maximal doses of PTZ-induced seizures. Furthermore, brivaracetam showed a higher than levetiracetam activity against secondarily generalized motor seizures in corneally kindled mice and hippocampal-kindled rats. Brivaracetam was also more effective than levetiracetam in suppressing clonic convulsions in audiogenic seizure-susceptible mice and spontaneous spike-and-wave discharges in genetic absence epilepsy rats [66]. Brivaracetam differs markedly from levetiracetam by its distinct interaction with SV2A, higher lipophilicity, faster brain penetration, and a more profound anti-seizure activity in animal models including amygdalar kindling in mice [67]. Racetams have been also tested in models of genetic epilepsy such as genetically sound-sensitive mice derived from a DBA strain and in the Genetic Absence Epilepsy Rats from the Strasbourg (GAERS) strain. Padsevonil provided potent, dose-dependent protection against seizures induced in sound-sensitive mice, a genetic model of generalized epilepsy. Brivaracetam was also active in this model, while levetiracetam showed a lower potency, correlating with their SV2A binding affinity [48, 66] In the GAERS model which is considered predictive of human absence epilepsy, padsevonil showed a higher potency than brivaracetam, and levetiracetam had a weak effect in this model. It is noteworthy that these drugs suppressed spontaneous spike-wave discharges (SWDs) in doses correlating with their affinity for SV2A [48, 66, 68]. Of new genetic models, recent data showed that levetiracetam significantly reduced ECoG spike frequency medication in a mouse model of potassium/sodium hyperpolarization-activated cyclic nucleotide-gated channel 1 (HCN1) developmental and epileptic encephalopathy [69].

**Table 1:** Anticonvulsant potencies of levetiracetam, brivaracetam, gabapentin and pregabalin in the commonly used acute experimental seizure models in mice

ASM \ Model	MES	PTZ	6 Hz (32 mA)	6 Hz (44 mA)
Levetiracetam	N.A.	N.A.	14.42a	345.4b
Brivaracetam	113c	30c	4.4d	N.D.
Gabapentin	> 400f	199.3e	72.11a	N.D.
Pregabalin	130.3f	N.D.	31.66a	N.D.

Data are presented as median effective doses (ED50 values in mg/kg) of the ASMs. MES– maximal electroshock-induced generalized tonic seizures; PTZ– pentylenetetrazole-induced generalized clonic seizures; 6 Hz–6 Hz corneal stimulation-induced secondary generalized motor seizures; N.A.– not active; N.D.– not determined. a– results from [77]; b– results from [167]; c– results from [66]; d– results from [168]; e– results from [133]; f– results from [130]

### Interaction profile of levetiracetam when combined with other ASMs– a preclinical perspective

Levetiracetam is considered to be “virtually ineffective” in the acute mouse generalized tonic-clonic (MES) and clonic (PTZ-induced) seizure models **(**Table 1**).** However, based on type II isobolographic analysis, it was found that levetiracetam produced either synergistic or additive interactions with carbamazepine, oxcarbazepine, topiramate, felbamate, and retigabine in the mouse MES model [70–72]. Levetiracetam produced also additive interactions with phenobarbital, valproate, phenytoin, lamotrigine, and pregabalin in the mouse MES model [70, 72]. In the mouse PTZ-induced seizure model, levetiracetam combined with gabapentin, clonazepam, ethosuximide, phenobarbital, and valproate produced synergistic or additive interactions, depending on the proportions of the ASMs used in the mixtures. The additive interactions were observed for the combinations of levetiracetam with tiagabine and vigabatrin in the mouse PTZ-induced seizure model [73, 74]. On the other hand, experimental evidence indicates that levetiracetam is fully effective in the mouse 6 Hz corneal stimulation-induced secondarily generalized motor seizure model (Table 1).

In this seizure model, the type I isobolographic analysis revealed that levetiracetam combined with lacosamide [75], phenobarbital [76], gabapentin, pregabalin, and retigabine [77] evoked supra-additive (synergistic) interactions. Levetiracetam combined with clonazepam, oxcarbazepine, tiagabine, and valproate triggered additive interactions in the mouse 6 Hz corneal stimulation-induced (32 mA) seizure model [76]. Additionally, levetiracetam in combination with the galanin agonist (810-2) produced supra-additive (synergistic) interaction in the mouse 6 Hz corneal stimulation-induced (32 mA) seizure model [78]. Levetiracetam in combination with JNJ-46,356,479, JNJ-42,153,605, JNJ-40,411,813, and LY-404,039 (four positive allosteric modulators of the metabotropic glutamate receptor subtype 2) resulted in supra-additive interactions in the mouse 6 Hz corneal stimulation-induced (44 mA) seizure model [79, 80]. Although neither the galanin agonist nor the positive allosteric modulators of the metabotropic glutamate receptor subtype 2 are ASMs, the interactions of levetiracetam with these agents were assessed using the isobolographic analysis of interaction. This was the reason for including the results of these experiments in the present review to attract attention to novel potentially effective drug candidates with unique molecular mechanisms of action, especially if these combinations evoked synergistic interactions in preclinical studies.

Considering the interaction profile of levetiracetam, one can ascertain that when combined with various ASMs, the drug triggered either synergistic or additive interactions in various acute models of experimentally evoked seizures in rodents. No antagonistic interactions between levetiracetam and ASMs were detected while using the isobolographic analysis of interactions.

The isobolographic analysis of interactions is the best method employed in pharmacological and toxicological studies to classify the pharmacodynamic interactions between ASMs [81, 82]. More detailed information on type I and II isobolographic analyses used in experimental epileptology can be found elsewhere [83].

In this review, we assessed and described only the interactions evoked by levetiracetam, gabapentin, and pregabalin with the currently available ASMs, based on the aforementioned type I and II isobolographic methods. Preclinical experiments assessing the effect of other drugs or compounds on the anticonvulsant potencies of the three ASMs (levetiracetam, gabapentin, and pregabalin) evaluated with a subthreshold method or other methods, which neglected the effects triggered by the tested compounds, were not considered herein.

### Clinical use of racetams in the treatment of epilepsy

Racetams are a class of drugs that share a pyrrolidone nucleus but possess diverse pharmacological activities and no well-defined mechanism of action. Of them, piracetam, aniracetam, oxiracetam, pramiracetam and phenylpiracetam show procognitive properties via positive allosteric modulation of the AMPA receptors, while levetiracetam, brivaracetam, and seletracetam belong to ASMs [84] [Löscher and Richter, 2000]. Levetiracetam is an ASM approved for the treatment of focal and generalized epilepsy, both in adults and children. The recommended dose range in adults is 1000–3000 mg/day. The advantages of levetiracetam are non-hepatic metabolism and lack of major interactions with other drugs which makes it a suitable medication for the elderly [85]. Along with lamotrigine and oxcarbazepine, it is also one of the safest ASMs used in the treatment of women at the childbearing age, with the potential teratogenic effect within the range of the population risk of major congenital defects [86–88]. Levetiracetam is also recommended in the treatment of status epilepticus due to its availability in the intravenous formula [5–7]. The limitations of therapy with levetiracetam are serious psychiatric side effects which can occur in about 13–30% of patients treated with this drug. The side effect spectrum consists of irritability and aggression and can be aggravated by psychosis, depression, and suicidal ideation [92]. The mechanism that can underlie these effects might be related to the levetiracetam impact on the AMPA receptor [93]. It has also been proven that the effectiveness of levetiracetam is lower than that of valproate in the treatment of idiopathic generalized epilepsies [94] and lower than lamotrigine in the treatment of focal epilepsies [95], which puts into question its use as a first-line drug in the treatment of epilepsy [96]. However, levetiracetam is currently widely used both as the first-choice drug or add-on therapy for the treatment of focal seizures, myoclonias, and generalized tonic-clonic seizures [97].

Brivaracetam has been approved for the treatment of focal seizures in adults and children ≥ 4 years of age, with an effective dose of 50–200 mg/day in the treatment of adults [98]. According to the US Food and Drug Administration (FDA), this indication includes monotherapy and adjunctive therapy, although brivaracetam has not been specifically evaluated in clinical trials for initial monotherapy and is only registered in add-on therapy in Europe [97]. Brivaracetam also proves to be effective in generalized epilepsy; however, it is used off-label in keeping with this indication [99]. It is metabolized in the liver and therefore its maximal dose has to be reduced by 1/3 in patients with liver impairment. It has a low potential for clinically relevant drug-drug interactions and can be used in the elderly [98]. Brivaracetam is available in an intravenous formula; which is why it can be a promising new option for the treatment of status epilepticus in the future [100]. The post-hoc results of clinical studies suggest that brivaracetam may be effective in the treatment of epilepsy in patients in whom treatment with levetiracetam has failed [101]. However, brivaracetam does not seem to be as effective in the treatment of epilepsy as it was previously thought, taking into consideration its superiority over levetiracetam in experimental studies [102, 103], where it was 15–30 times more potently bound to the SV2A molecule than levetiracetam and it entered the brain much faster due to its lipophilic properties [104]. Nevertheless, brivaracetam seems to have a better safety profile allowing for the use of the medication in the treatment of patients in which levetiracetam had to be discontinued due to psychiatric side effects [92, 105]. The optimal scheme in which the drugs should be replaced is not clear; however, the most frequently used ratio is 10:1 (for example, 1000 mg of levetiracetam is the equivalent of 100 mg of brivaracetam) or 15:1 [103, 106]. The results of observational studies suggest that this change can be done overnight without fearing new side effects development or deterioration of the previously experienced side effects [102, 103, 107]. Treatment with brivaracetam can, however, result in adverse events similar to these observed while administering levetiracetam, among them are somnolence, fatigue, irritability, dizziness, insomnia, anxiety, and depression [98]. Despite close structural similarities, various racetams have different pharmacological and clinical profiles [108]. Although piracetam was marketed 50 years ago, its mechanism of action is still poorly understood. Originally marketed by UCB Pharma in 1971, piracetam was the first nootropic drug to modulate cognitive function without causing sedation or psychostimulation. The mechanism presumably comprises the restoration of cell membrane fluidity in elderly animals and humans, modulation of cholinergic and glutamatergic transmission, improvement of neuroplasticity, and facilitation of microcirculation [109]. Levetiracetam and other S-enantiomers, but not piracetam, show a high affinity to a brain-specific stereoselective binding site, later described as SV2A protein. Preclinical studies revealed that piracetam is more efficient in improving learning and memory, while levetiracetam shows a much higher efficacy than piracetam in preventing seizures. Piracetam has a moderate or no inhibitory effect on generalized tonic or clonic seizures. Still, it reduces the incidence and duration of spike-wave discharges in the model of absence seizures and the number of spikes/min in the cobalt-induced focal epilepsy model. Despite its negligible effects in most screening tests, it can significantly improve the efficiency of other ASMs via non-pharmacokinetic interactions [110]. Clinically, piracetam is mainly used in the treatment of post-stroke aphasia and age-related cognitive disorders, vertigo, dyslexia, and sickle cell anemia, and in very high doses it is also effective against cortical myoclonus [111]. On the other hand, levetiracetam is a widely used ASM in focal-onset, myoclonic and generalized tonic-clonic seizures. Further studies have supported the notion that piracetam-derived drugs can be roughly divided into two categories– cognitive enhancers and ASMs. To this end, a systematic review of the pharmacology of piracetam and piracetam-related drugs revealed that piracetam, oxiracetam, aniracetam, pramiracetam, and phenylpiracetam were used in the treatment of various cognitive impairments; this group of medications, phenylpiracetam was used for a wider range of indications showing more potency than piracetam [112]. Other piracetam-related drugs such as levetiracetam, seletracetam, and brivaracetam demonstrated antiepileptic activity, whereas their procognitive effects remained unclear. Although SV2A is regarded as the primary molecular target for antiseizure effects of levetiracetam and brivaracetam, new ligands of SV2A have been designed that show procognitive but not antiseizure properties [113]. Very recently, a highly selective SV2A ligand SDI-118 devoid of anticonvulsant activity was reported to display significant cognitive-enhancing effects in various animal tests and the first-in-human randomized controlled trial [114]. Overall, elucidation of the intriguing contribution of SV2A in anticonvulsive and/or procognitive effects of racetams warrants further studies.

### Neurochemical mechanisms of gabapentin and pregabalin

Gabapentin has been synthesized as an analog of GABA, which is an inhibitory neurotransmitter in the central nervous system. Gabapentin, however, does not show any action similar to GABA or does not evoke any effect on the GABA receptors [115]. The mechanism of action of gabapentin consists of suppressing the subunit α2δ1 of the voltage-gated calcium channel, which results in the inhibition of calcium influx into the neuron and decreases its excitability. Pregabalin was synthesized in 2004 as a drug similar to gabapentin in its pharmacodynamics, but more potent in the suppression of the subunit α2δ1 of the voltage-gated calcium channel. The abundantly expressed in the nervous system membrane-anchored extracellular α2δ glycoproteins form auxiliary subunits of the voltage-gated calcium channels, which are essential components of excitable cells and, among others, regulate presynaptic neurotransmitter release. Mutations and polymorphisms of human (CACNA2D1-4) genes encoding for the four known α2δ proteins (isoforms α2δ1 to α2δ4) are linked with several central nervous system disorders including epilepsy [116, 117]. Using the cellular α2δ subunit triple-knockout/knockdown model, it has been demonstrated that the α2δ subunits play a crucial role in the formation and organization of glutamatergic synapses, especially in the presynaptic differentiation. There is also convincing research evidence that the α2δ subunits may be engaged in critical steps during synapse maturation [118]. Both α2δ1 and α2δ2 are the targets for gabapentin and pregabalin. Interestingly, when administered acutely, these drugs produce only a slight inhibition of the calcium channel currents. In contrast, their chronic administration inhibits the currents and impairs the trafficking of the α2δ subunits resulting in the inhibition of synaptic transmission [119]. Recent reports indicate that α2δ1 protein can interact not only with the voltage-dependent calcium channels but also with other presynaptic proteins, including the NMDAR, neurexin-1α and adhesion molecules (thrombospondins) **(**Table 2**)** It has been suggested that these interactions may contribute to the mechanisms of gabapentinoids therapeutic effects in neuropathic pain [120]. Other investigators speculated that the thrombospondin/α2 δ axis is important for the correct functioning of the cortico-thalamo-cortical circuits and that disturbances in this axis may contribute to the pathomechanisms of absence epilepsy [Celli et al., 2017]. AMPARs are complexes composed of glutamate A1 (GluA1), GluA2, GluA3, and GluA4 subunits which form functionally different heterotetramers [7]. Li et al. (2021) demonstrated that α2δ1 is a key AMPA receptor-interacting protein that controls the subunit composition and Ca^2+^ permeability of the postsynaptic AMPA receptors. They reported that α2δ1 inhibits the glutamate GluA1/GluA2 heteromeric assembly and increases the GluA2 retention in the endoplasmic reticulum, and these effects can be reversed by gabapentin. These results point to an important role of α2δ1 in modulating synaptic functions because the AMPA receptors which lack the GluA2 subunits are Ca^2+^-permeable and their presence is linked with some neurologic disorders [121]. Zhou et al. (2021) showed the interdependence of α2δ-1 and PKC phosphorylation in regulating synaptic trafficking and activity of NMDAR. Via the inhibition of α2δ1, pregabalin interferes with the phosphorylation of NMDAR, in this way affecting neuroplasticity, which, among others, is the key element of epileptogenesis [122]. Gabapentin dose-dependently reduced neuronal injury induced by cerebral ischemia-reperfusion preventing the oxidative stress-related autophagy via the activation of the phosphoinositide 3 kinase (PI3K)/Akt/mammalian (or mechanistic) target of rapamycin (mTOR) (PI3K/Akt/mTOR) pathway [123]. Of note, both the oxidative stress and PI3K/Akt/mTOR pathway are also linked with molecular mechanisms of epileptogenesis [124]. **(**Table 2**).**

**Table 2:** The α2δ1-mediated effects of gabapentinoids

Molecular target	Mechanism	Effect	References
VGCCs	inhibition of calcium influx into the neuron and decreases its excitability	anti-seizures	[115]
presynaptic proteins engaged in the organization of glutamatergic synapses	interference with the formation and maturation of glutamatergic synapses	anti-epileptogenic?	[118]
neurexin-1α, and adhesion molecules (thrombospondins)	inhibition of aberrant excitatory synaptogenesis	analgesia, detrimental effect in the absence epilepsy	[8, 120, 122]
AMPAR	controlling the subunit composition and Ca^2+^ permeability of postsynaptic AMPARs	analgesia	[121]
NMDAR	interference with the phosphorylation of NMDAR, in this way, affecting neuroplasticity	anti-epileptogenic?	[122]
PI3K/Akt/mTOR pathway	preventing the oxidative stress-related autophagy via the activation of the PI3K/Akt/mTOR pathway	neuroprotective, anti-epileptogenic?	[123]

VGCCs - voltage-gated Ca2 + channels, AMPAR - α-amino-3-hydroxy-5-methyl-4-isoxazolepropionic acid receptor, NMDAR– N-methyl-D-aspartate receptor, PI3K/Akt/mTOR - the phosphoinositide 3 kinase (PI3K)/Akt/mammalian (or mechanistic) target of the rapamycin (mTOR) pathway

The above-mentioned molecular mechanisms suggest that gabapentinoids may have modifying effects on the course of epilepsy development. Beyond the α2δ1 binding sites, gabapentin and pregabalin may show different affinities to other neuronal molecular targets. Thus, it was reported that gabapentin is a potent activator of the heteromeric KCNQ2/3 voltage-gated potassium channel and the homomeric KCNQ3 and KCNQ5 channels, whereas pregabalin at higher concentrations displays opposite effects [125].

### Pharmacological profiles of gabapentin and pregabalin in rodent models of seizures and epilepsy

Gabapentin and pregabalin show similar, but not identical pharmacological profiles in animal seizure models. Both drugs display anti-seizure activity in primary generalized tonic-clonic seizures (maximal electroshock seizure threshold test [MEST]– gabapentin [126] and MES– pregabalin–focal seizures (6-Hz test; 32 or 44 mA), and focal seizures in kindling. They are inactive in the genetic models of absence seizures (GAERS or WAG/Rij rat strains). Gabapentin has been reported to decrease the duration of hyperthermia-induced seizures in Scn1a mutant rats [127] but had no significant effect on seizure frequency in the lethargic (lh/lh) mouse model of absence seizures [128].

### Interaction profile of gabapentin when combined with other ASMs– a preclinical perspective

**Fig. 2:**
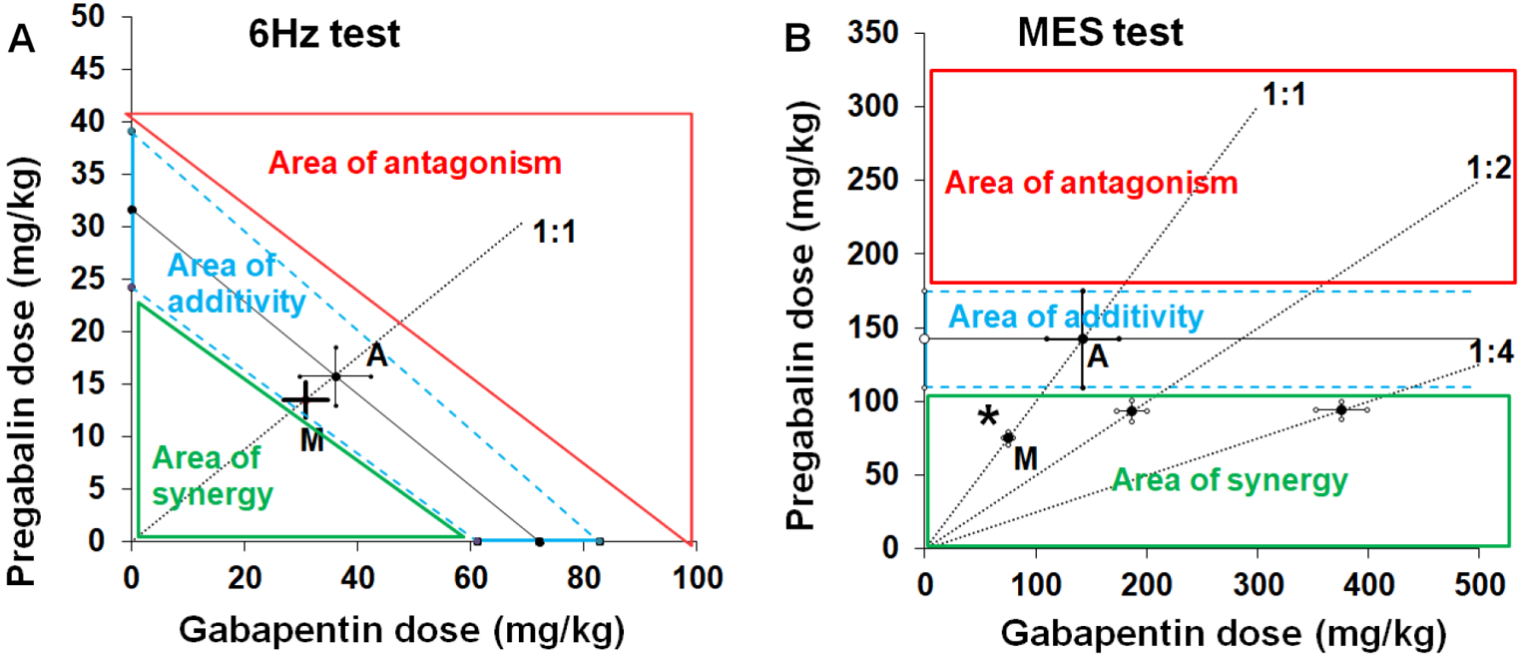
Isobolograms illustrating interactions between gabapentin and pregabalin in the 6 Hz corneal stimulation-induced seizure (**A**) and maximal electroshock-induced (MES) seizure (**B**) tests in mice. **A.** The type I isobolographic analysis was used to assess the interactions between two fully effective drugs in the mouse 6 Hz test (**A**). The isobologram for the combination of two fully active drugs, pregabalin and gabapentin in the 6 Hz test was presented earlier [77], and partly modified to display isobolographically areas of synergy, additivity and antagonism, where the experimentally derived ED50 mix value can be placed. The ED50 values for pregabalin and gabapentin when used alone in the 6 Hz test are placed on the ordinate (Y) and abscissa (X) of the Cartesian plot system of coordinates, respectively. The line connecting these ED50 values on the X and Y axes is the line of additivity. The diagonal line starting from the beginning of the Cartesian plot system and crossing the line of additivity indicates the proportion of the two-drug mixture (at the fixed-ratio of 1:1). For the interaction of pregabalin with gabapentin in the 6 Hz test, the experimentally derived ED50 mix (as the point M on the graph) is placed in the area of additivity, close to the point A, which reflects the theoretically calculated ED50 add value, predicted to exert additive interaction between drugs. **B**. The type II isobolographic analysis was conducted to evaluate the interaction between drugs of which gabapentin was ineffective in the mouse MES test (**B**). The isobologram for the combination of pregabalin (a fully active drug) and gabapentin (a virtually ineffective drug) in the MES test was presented earlier [130], and partly modified to display isobolographically areas of synergy, additivity and antagonism, where the experimentally derived ED50 mix value can be placed. The ED50 value for pregabalin when used alone in the mouse MES test is placed on the ordinate of the Cartesian plot system of coordinates. The line parallel to X axis starting from the ED50 for pregabalin is the line of indifference (additivity). The diagonal lines starting from the beginning of the Cartesian plot system and crossing the line of additivity indicate the proportions of the drugs in the mixture (at the fixed-ratios of 1:1, 1:2 and 1:4). For the interaction of pregabalin with gabapentin in the MES test, the experimentally derived ED50 mix (as the point M on the graph for the fixed-ratio of 1:1) is placed in the area of synergy, significantly below the area of additivity (*p < 0.05), which reflects the theoretically calculated ED50 add value, predicted to exert additive interaction between drugs

Gabapentin is considered to be “virtually ineffective” in the mouse MES model, but its interaction profile with other ASMs in this seizure model was determined with type II isobolographic analysis **(**Table 1**).** Preclinical studies provided evidence that gabapentin interacted both synergistically and additively with commonly used ASMs (including carbamazepine, phenytoin, phenobarbital, lamotrigine, valproate, oxcarbazepine, topiramate, tiagabine, and talampanel) in the mouse MES model [126, 129]. Because both pregabalin and gabapentin are characterized by similar molecular mechanisms of action, it was surprising to observe that the combination of pregabalin with gabapentin triggered a synergistic interaction in the mouse MES model with type II isobolographic analysis (Fig. 2B) [130], and simultaneously, an additive interaction in the mouse 6 Hz corneal stimulation-induced (32 mA) seizure model with type I isobolographic analysis (Fig. 2A) [77]. In the mouse PTZ-induced seizure model, gabapentin produced synergistic interactions with vigabatrin [131] and oxcarbazepine [132], and an additive interaction with tiagabine [133], felbamate, and loreclezole [134]. In the mouse 6 Hz corneal stimulation-induced (32 mA) seizure model, gabapentin combined with lacosamide triggered either a supra-additive interaction or an additive interaction [77]. Gabapentin combined with levetiracetam or retigabine triggered supra-additive interactions in the mouse 6 Hz corneal stimulation-induced (32 mA) seizure model [77]. (Fig. 2A-1B**).**

### Interaction profile of pregabalin when combined with other ASMs– a preclinical perspective

Pregabalin evoked additive interactions with phenobarbital, oxcarbazepine, lamotrigine, topiramate, carbamazepine, valproate, and levetiracetam in the mouse MES model [135–138]. Pregabalin combined with lacosamide, levetiracetam, or retigabine triggered synergistic interactions in the mouse 6 Hz corneal stimulation-induced (32 mA) seizure model [77]. Pregabalin combined with gabapentin produced an additive interaction in the mouse 6 Hz corneal stimulation-induced (32 mA) seizure model [77], but the same two-drug combination triggered a synergistic interaction in the mouse MES model [130].

### Interaction profile of pregabalin when combined in three-drug mixtures– a preclinical perspective

In contrast to several two-drug combinations, only a few three-drug combinations were evaluated experimentally in the mouse MES model. Experimental evidence indicates that the combination of pregabalin with phenobarbital and phenytoin (with a fixed ratio of 1:1:1) produced synergy in the mouse MES model [139]. Similarly, in the case of the combinations of pregabalin with phenobarbital and lamotrigine, pregabalin with phenobarbital and oxcarbazepine, and pregabalin with phenobarbital and topiramate [140], pregabalin with lacosamide and topiramate, pregabalin with lacosamide and oxcarbazepine, pregabalin with oxcarbazepine and topiramate [141], all the six combinations evoked synergistic interactions in the mouse MES model. Only the combination of pregabalin with lacosamide and lamotrigine produced an additive interaction in the mouse MES model [141]. To date, no other three-drug combinations of ASMs, containing levetiracetam and/or gabapentin have been tested experimentally in the animal seizure models.

### Clinical use of gabapentinoids in the treatment of epilepsy

Gabapentin is a narrow-spectrum ASM approved for the treatment of focal epilepsy as monotherapy or adjunctive therapy in adults and children ≥ 12 years and in the treatment of neuropathic pain. The recommended dose in adults is 900–3600 mg/day. Gabapentin is also used off-label for generalized anxiety disorder and panic disorder, obsessive-compulsive disorder, addictions, bipolar disorder, essential tremor, and migraine prophylaxis [142, 143]. The medication is approved in Europe for initial monotherapy [97]. However, in a large comparative study, gabapentin was found to be less effective than lamotrigine [144]. The distribution of the drug and its non-linear absorption pharmacokinetics requires its dosing three times daily [145].

Pregabalin is indicated as the adjunctive therapy in epilepsy with focal seizures in adults. The recommended dose is 300–600 mg/day [146]. Other indications include neuropathic pain and generalized anxiety disorders. Off-label indications are similar to those of gabapentin [142]. Pregabalin is also used in the treatment of symptoms of fibromyalgia [147]. In focal seizures, pregabalin has been found to be less beneficial than lamotrigine as the first-choice drug [148] and probably should not be used as the first-line ASM [97]. However, the results of the study in which the conversion of previous treatment to pregabalin monotherapy was assessed were favorable for the drug [149]. Pregabalin shows more beneficial pharmacokinetics than gabapentin. The oral availability of pregabalin (90%) is higher than that of gabapentin (60%) and its pharmacokinetics is linear which allows for its use in a twice-daily regimen. The conversion of treatment from gabapentin to pregabalin may increase adherence to therapy, as it is usually more convenient for the patients to take their medications twice daily instead of three times per day. The scheme of drug replacement is 6:1, where a gabapentin daily dose of 1800 mg is the equivalent of a 300 mg dose of pregabalin [150].

Gabapentinoids (gabapentin and pregabalin) do not provoke any major drug-drug interactions. They are usually well tolerated; however, the side effects that may occur during treatment are somnolence, dizziness, weight gain, and peripheral edema [146]. Treatment with gabapentinoids also poses a risk of abuse and dependence, especially in patients abusing other drugs and substances in the past [142, 146]. Gabapentinoids can also pose a serious risk for respiratory depression, especially in patients using opioids subjects with pulmonary diseases, or in the elderly [150].

### Drug combinations including levetiracetam, pregabalin, or gabapentin in therapy of epilepsy– a clinical perspective

If the treatment of epilepsy with the first ASM fails due to its ineffectiveness, the substitution monotherapy or adjunctive therapy options are equivalent. Adjunctive therapy for epilepsy is preferred when the first antiepileptic drug is well tolerated and only partially effective, or the drug to be added to the therapy has not been tested in monotherapy [151]. The adjuvant drug should not have any adverse pharmacokinetic interactions with the first antiepileptic medication or other concomitant drugs [152]. Rational polytherapy should maximize the efficacy and minimize the side effects [153]. The presented scientific data indicate that the combination of two ASMs with different mechanisms of action is better in balancing tolerance and effectiveness, and the combination of drugs with similar mechanisms is associated with increased side effects [154]. In human clinical trials, the synergistic effect of only one combination of lamotrigine and valproate has been confirmed [155, 156]. Regardless of the rather limited use of gabapentin and the widespread use of racetams in the treatment of epilepsy, medications from both these therapeutic groups are an important element of drug combinations in drug-resistant epilepsy. This is facilitated by the fact that representatives of both these groups are characterized by the lowest interaction potential among all ASMs [157].

Clinical studies indicated that the two-drug combination of gabapentin with carbamazepine produced seizure freedom in 37 patients with focal and generalized seizures [158, 159]. Additionally, the combinations of levetiracetam with carbamazepine, levetiracetam with lamotrigine, and levetiracetam with valproate successfully protected 23, 19, and 16 patients with focal and generalized seizures, respectively [159]. The most successful triple therapy regimens were those containing the combinations of levetiracetam with lamotrigine and valproate, lamotrigine with carbamazepine or lamotrigine with topiramate [159] or gabapentin with carbamazepine and topiramate [158].

### Clinical possibilities for evaluating drug combinations of ASMs

**Fig. 3:**
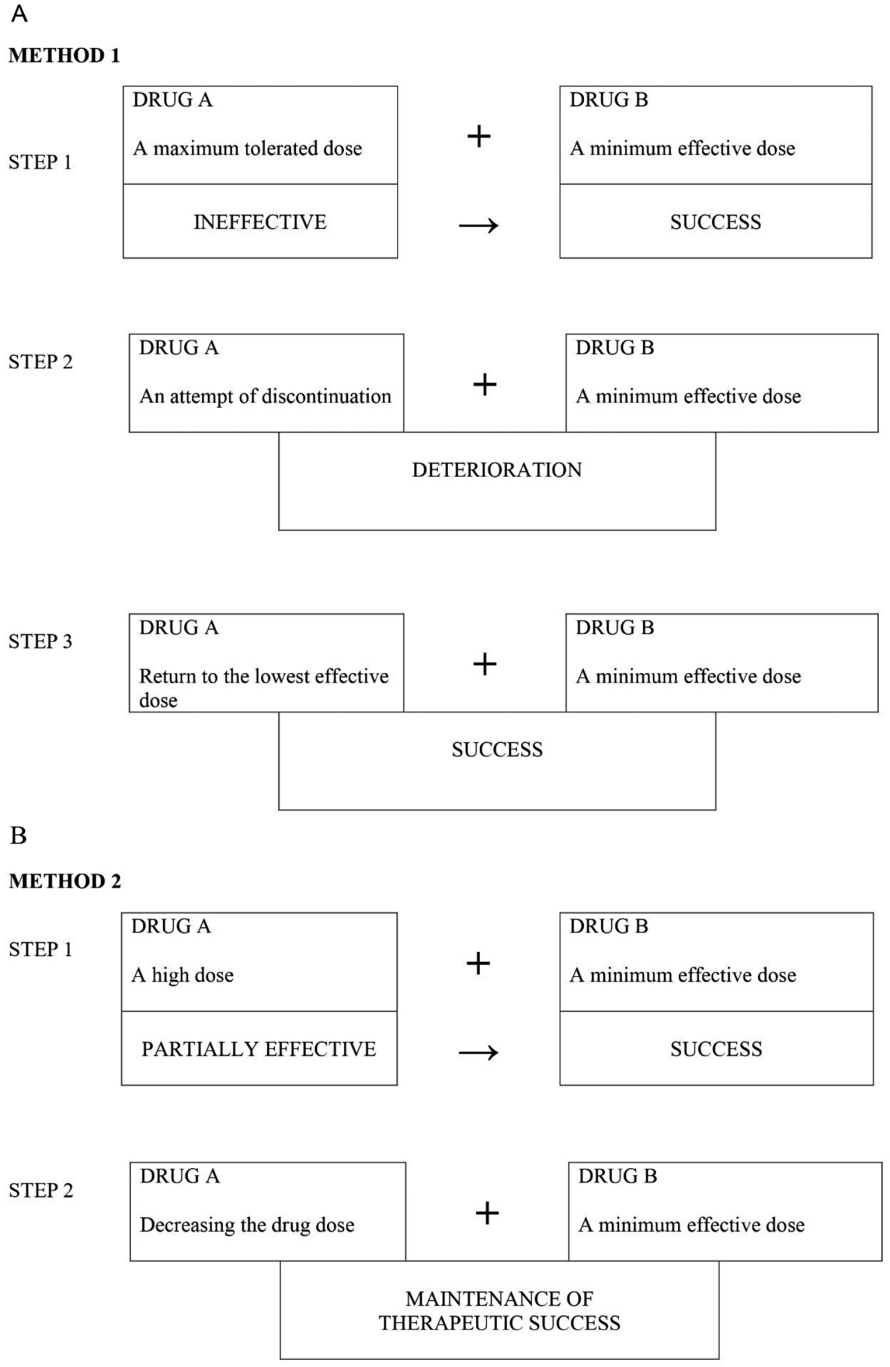
Two proposed methods of clinical evaluation of drug combinations.**A**. Method 1 assumes the ineffectiveness of drug A at the maximum tolerated dose and a therapeutic success after adding the minimum effective dose of drug B (first step), an unsuccessful attempt at discontinuing drug A (second step) and a repeated therapeutic success after reinstating drug A but at the minimum effective dose (third step). **B**. Method 2 assumes a partial effectiveness of drug A used at a high dose and a therapeutic success after adding a minimal effective dose of drug B (first step) and the maintenance of the said effect after lowering the dose of the first drug without attempting to discontinue its administration (step two)

A clinical method of assessing the value of a drug combination was proposed by Stephen et al. [160]. The method evaluated the effectiveness of a combination of drug A and drug B in a situation where previously neither drug A nor drug B were effective in monotherapy when administered in maximum tolerated doses. Czapinski et al. (2014) proposed two other methods of clinical evaluation of the effectiveness of drug combinations (Fig. 3AB).

An antagonistic combination in terms of adverse effects was considered when the result obtained before using the second medication was higher than after adding the second drug. Based on the proposed research method Czapinski et al. observed that the combination of levetiracetam + lamotrigine was synergistic in effectiveness and antagonistic in adverse symptoms and that the combination of topiramate + levetiracetam was synergistic in effectiveness and antagonistic in adverse symptoms in patients with focal impaired awareness automatism seizures progressing to bilateral tonic-clonic seizures. Furthermore, the combination of oxcarbazepine + levetiracetam was synergistic in effectiveness and antagonistic in adverse symptoms. Employing the above method, Czapinski et al. have found clinical evidence of the efficacy synergism and adverse symptoms antagonism in several combinations including gabapentin or levetiracetam: levetiracetam + lamotrigine, topiramate + levetiracetam, levetiracetam + oxcarbazepine, gabapentin + lamotrigine [147].

## Discussion

It appears that combining ASMs with various mechanisms of action is more efficacious than using combinations of ASMs sharing the same or comparable mechanisms [161]. The results from combinations of gabapentin (or pregabalin) with other ASMs seem to support this assumption. For instance, synergistic combined treatments of gabapentin with carbamazepine, valproate, phenytoin, oxcarbazepine, topiramate, lamotrigine, or tiagabine involve ASMs affecting various brain targets [161]. Also, levetiracetam, in certain dose ratios, proved to be synergistic when combined with carbamazepine, oxcarbazepine, topiramate, felbamate, and retigabine. The same conclusion may be drawn when analyzing 3-ASM combinations. Considering the 6-Hz seizure model, synergistic interactions of levetiracetam with numerous ASMs, including gabapentinoids, need to be accentuated. Furthermore, the lack of synergy shown by the combined treatment of gabapentin + pregabalin in this test may be explained based on their identical mechanisms of action. However, at one fixed dose ratio, both the ASMs combined evoked a synergistic anticonvulsant interaction which is, however, not further seen at different dose ratios in the MES test. At present, there is no good explanation for this surprisingly beneficial interaction among these ASMs in this seizure model.

There is a good correlation between the experimental and clinical data on the nature of interactions among ASMs. Practically, only one contradictory result exists regarding the combined treatment of carbamazepine + lamotrigine shown as antagonistic by isobolography and claimed as positive from the clinical point of view [158]. This discrepancy probably arises from a different understanding of antagonism in experimental studies which may be more demanding. This problem has been discussed in detail in a review by Błaszczyk et al. (2018) [162]. Anyway, all the positive combinations of ASMs derived from experimental studies are highly likely to be clinically efficient although several limitations must be taken into consideration. In fact, among the undesired effects, only neurotoxicity was considered, and some other peripheral untoward actions might have been missed. Moreover, the experimental data were obtained after acute injections of ASMs, so a possibility thus arises that in clinical conditions more pharmacokinetic events may be encountered in case of a chronic drug administration. After all, the experiments were carried out on non-epileptic animals, so the results were dependent on the normal brain. Nevertheless, experimental convulsions induced in non-epileptic rodents may be ascribed to different seizure models– for instance, the results from MES may have the best predictive value for generalized tonic-clonic seizures [163], from the PTZ test– for myoclonic and to a certain degree for absences [164], and from 6-Hz– for drug-resistant focal seizures [165]. In light of the 6-Hz test, all synergistic 3-drug combinations need to be especially underlined.

## Conclusions

Summing up, preclinical data indicate that gabapentinoids and levetiracetam combined with many other ASMs exhibit a synergistic anticonvulsant activity with generally low neurotoxic potential. Clinical data, although obtained from a limited number of patients, seem to support preclinical findings. Knowledge of the ASMs mechanism of action may guide add-on or substitution decisions but is of limited predictive value for the efficacy and tolerability of these drugs. Therefore, developing a better understanding of ASM synergy could lead to considerably better outcomes for patients, and could enable them to avoid some of the ‘trial and error’ effects of different drug combinations that many patients currently undergo. The beneficial pharmacological profile of racetams and gabapentinoids suggests that the presynaptic release machinery appears to be a particularly promising target for further ASMs.

### Future directions

Recent decades have witnessed spectacular progress in better understanding the pathophysiology of epilepsy and potential molecular targets for pharmacotherapy of the heterogenous group disorders. However, the relationship between the mechanism of actions of ASMs and their clinical efficacies has been unraveled in some cases only. Likewise, the knowledge of the basic mechanisms of ASMs modestly, if at all, affects a clinician’s decision on polytherapy. The majority of screening tests identify chemical compounds that after single administration suppress acute seizures in rodents. Such compounds may limit the number of seizures but do not necessarily have disease-modifying properties. It is commonly accepted that pathological transformation of neuronal circuits underlies epilepsy, thus the future development of ASMs should be able to target neuroplastic processes and restore normal neurotransmission. As discussed in this article, the presynaptic mechanisms of some ASMs have been partly disclosed, but further studies are warranted for elucidating the interrelationships between the ASM’s molecular targets, e.g., the SV2A and α2δ1 proteins, via various presynaptic signaling pathways. Overall, presynaptic machinery remains still an unexplored and promising area for identifying new potential targets for new ASMs.

## Data Availability

Data sharing is not applicable to this article as no datasets were generated or analyzed during the current study. All authors have contributed equally to the work.

## Abbreviations

AMPAR: α-amino-3-hydroxy-5-methyl-4-isoxazolepropionic acid receptor
ASMs: Antiseizure Medications
DNM1: Dynamin 1
EAA: Excitatory Amino Acid
EPSCs: Excitatory Postsynaptic Currents
FDA: US Food and Drug Administration
GABA -γ: Aminobutyric Acid
GABA AR: GABA A Receptor
GABA-T: GABA Transaminase
GAERS: Genetic Absence Epilepsy Rats from the Strasbourg
GAT-1: GABA transporter 1
GPR55: G Protein-coupled Receptor 55
GTP: Guanosine Triphosphate
HCN1: Potassium/Sodium Hyperpolarization-activated Nyclic Nucleotide-gated channel 1
IK(DR): Delayed-Rectifier K + current
IK(M): Depolarization-Induced M-type K + current
INa: Voltage-gated Na + current
IP_3_R: 1,4,5-Trisphosphate Receptor
IPSCs: Inhibitory Postsynaptic Currents
KO: Knockout phenotype
LTP: Long-Term Potentiation
MES: Maximal Electroshock
mTORC1: mammalian Target Of Rapamycin Complex 1
NMDAR: N-Methyl-D-Aspartate Receptor
PI3K/Akt/Mtor: the phosphoinositide 3 kinase (PI3K)/Akt/mammalian (or mechanistic) target of the rapamycin (mTOR) pathway
PPAR-γ: Peroxisome Proliferator-Activated Receptor gamma
Prrt2: Presynaptic proline-rich transmembrane protein 2
PTZ: Pentylenetetrazol
SC: Sub Cutaneous
SVs: Synaptic Vesicles
SNAP25: Synaptosomal Associated Protein 25
SNARE: “SNAP REceptors”
v-SNARE: vesicle-associated SNARE
SV2A: Synaptic Vesicle protein type 2 A
STX7: Syntaxin 7
SWDs: Spontaneous spike-Wave Discharges
TRPV-1: Transient Receptor Potential Vanilloid 1 channel
VGCCs: Voltage-Gated Ca2 + Channels
VGSCs: Voltage-Gated Na + Channels
